# *δ*-Generalized Labeled Multi-Bernoulli Simultaneous Localization and Mapping with an Optimal Kernel-Based Particle Filtering Approach

**DOI:** 10.3390/s19102290

**Published:** 2019-05-17

**Authors:** Diluka Moratuwage, Martin Adams, Felipe Inostroza

**Affiliations:** 1 Department of Electrical Engineering & Advanced Mining Technology Center Universidad de Chile, 837-0451 Santiago, Chile; 2 Department of Electrical Engineering Universidad de Chile, 837-0451 Santiago, Chile; finostro@ug.uchile.cl

**Keywords:** SLAM, robotics, tracking, random finite sets

## Abstract

Under realistic environmental conditions, heuristic-based data association and map management routines often result in divergent map and trajectory estimates in robotic Simultaneous Localization And Mapping (SLAM). To address these issues, SLAM solutions have been proposed based on the Random Finite Set (RFS) framework, which models the map and measurements such that the usual requirements of external data association routines and map management heuristics can be circumvented and realistic sensor detection uncertainty can be taken into account. Rao–Blackwellized particle filter (RBPF)-based RFS SLAM solutions have been demonstrated using the Probability Hypothesis Density (PHD) filter and subsequently the Labeled Multi-Bernoulli (LMB) filter. In multi-target tracking, the LMB filter, which was introduced as an efficient approximation to the computationally expensive δ-Generalized LMB (δ-GLMB) filter, converts its representation of an LMB distribution to δ-GLMB form during the measurement update step. This not only results in a loss of information yielding inferior results (compared to the δ-GLMB filter) but also fails to take computational advantages in parallelized implementations possible with RBPF-based SLAM algorithms. Similar to state-of-the-art random vector-valued RBPF solutions such as FastSLAM and MH-FastSLAM, the performances of all RBPF-based SLAM algorithms based on the RFS framework also diverge from ground truth over time due to random sampling approaches, which only rely on control noise variance. Further, the methods lose particle diversity and diverge over time as a result of particle degeneracy. To alleviate this problem and further improve the quality of map estimates, a SLAM solution using an optimal kernel-based particle filter combined with an efficient variant of the δ-GLMB filter (δ-GLMB-SLAM) is presented. The performance of the proposed δ-GLMB-SLAM algorithm, referred to as δ-GLMB-SLAM2.0, was demonstrated using simulated datasets and a section of the publicly available KITTI dataset. The results suggest that even with a limited number of particles, δ-GLMB-SLAM2.0 outperforms state-of-the-art RBPF-based RFS SLAM algorithms.

## 1. Introduction

Simultaneous Localization and mapping (SLAM) is considered to be a fundamental process required by many mobile robotic applications. The SLAM process involves building a map of a prior unknown, unstructured environment using data from noisy exteroceptive and proprioceptive sensors mounted on a robot and estimating its position with respect to this map.

Over the years, many improvements to the estimation theoretic SLAM problem have been proposed since its inception in the seminal work of Smith, et al. [1]. An extended Kalman Filter-based SLAM (EKF-SLAM) solution was proposed by Dissanayake, et al. [2]. In [3], the FastSLAM algorithm was proposed as a factored solution to the SLAM problem, where the concept of Rao–Blackwellized particle filter (RBPF) was adopted. To reduce the computational cost of EKF-SLAM, an extended information filter-based SLAM algorithm, EIF-SLAM, was proposed in [4]. The SLAM problem has been solved as a maximum a posteriori (MAP) estimation problem by modeling it as a factor graph in recent years [5]. Graph SLAM [6] and Square Root Smoothing and Mapping (SAM) [7] are prime examples of MAP-based estimation. Although these methods generally produce superior results, their performance can be severely degraded due to the heuristic-based data association phase [8]. In addition, such solutions assume that the environment is static, which may produce inconsistent results in dynamic environments.

This article proposes that a map state estimation approach based on the random finite set (RFS) filtering framework can efficiently solve the data association problem in a principled manner within a Bayesian filtering framework while relaxing the static environment assumption. Traditionally, the robot position, the landmark map, and the measurements are represented as random vectors in both MAP-based optimization and Bayesian estimation theoretic approaches. In its random vector form, this representation requires the solution of additional sub-problems such as the determination of observation to feature associations, measurement clutter (false alarms) removal, and map management before the application of nonlinear optimization or Bayesian recursion. Parallel to the developments in SLAM, in multi-target tracking, Mahler [9] suggested the random finite set (RFS) representation of the multi-target state as opposed to random vector representation. He showed that, in contrast to the random vector representation, the RFS representation of a multi-object state offers a mathematically consistent notion of estimation error [9]. Furthermore, the uncertainties present in practical multi-target tracking applications, such as target detection and data association uncertainties as well as random clutter, are taken into account in the RFS representation of the multi-target state. As tractable approximations to the optimal Bayesian multi-target tracking solution, Mahler devised the Probability Hypothesis Density (PHD) filter, Cardinalized PHD (CPHD) filter and the multi-Bernoulli filter [9,10,11]. More recently, the δ-Generalized Labeled multi-Bernoulli (δ-GLMB) filter was proposed by Vo et al. as an analytical solution to the optimal multi-target Bayes filter [12,13]. In [14], the Labeled Multi-Bernoulli (LMB) filter was proposed by Reuter et al. as an efficient approximation to the computationally expensive δ-GLMB filter. Further, by combining the prediction and update steps and introducing a truncation approach using a Gibbs sampler, Vo et al. introduced an efficient implementation of the δ-GLMB filter in [15].

Due to the similarity between estimation theoretic SLAM problem and multi-target tracking, an RFS representation was adopted in SLAM by modeling the landmark map and observations as RFSs. The first RFS based SLAM solution was proposed by Mullane et al. [16] and later they proposed PHD-SLAM, an improved SLAM solution using PHD filter-based mapping and Rao–Blackwellized particle filter-based trajectory estimation [17,18]. Lee et al. also demonstrated a PHD-SLAM algorithm by modeling the SLAM problem as a single cluster multi-object model [19]. Despite modeling the landmark map as a Poisson distributed RFS, the PHD-SLAM algorithm produces robust results against noisy sensory information and measurement clutter. In [20], Deusch et al. proposed LMB-SLAM as an improved solution to the SLAM problem, by representing a landmark not only by its kinematic state but also by a label and propagating the landmark map using an LMB filter. Filter-based SLAM solutions with semantic features may benefit from such a representation for associating the features to physical objects in the environment. Furthermore, SLAM solutions with multiple feature types may benefit from a labeled representation due to convenience in identification and classification (see [21] for an example SLAM algorithm that may benefit from labeled feature models).

LMB-SLAM, which makes no assumptions about the map, in general, produces superior results compared to PHD-SLAM, which models the landmark map as a Poisson distributed RFS. However, during the measurement update, the LMB filter first converts its LMB distribution into a δ-GLMB distribution, performs the measurement update, and then converts the δ-GLMB distribution back to an LMB distribution. In general, this process yields a loss of information and degrades performance (see [22] for comparisons). Moreover, the performance of the LMB-SLAM filter depends on the underlying LMB filter’s ability to partition the landmark measurements and estimated landmark tracks in the sensor Field Of View (FOV) into spatially independent clusters and update them in parallel during the measurement update step [22]. If the measurements and tracks are easily separable into such independent groups (clusters), the LMB filter performs efficiently. However, if this is not possible, the computational cost is equivalent to the δ-GLMB filter. Although closely spaced targets are uncommon in multi-target tracking applications, in SLAM, it is common to encounter clustered and unevenly distributed landmarks which appear in the sensor FOV. Depending on the type of sensor, resolution, and the algorithms used, some feature extraction methods may also produce multiple closely spaced features, which may degrade performance in LMB-SLAM. Furthermore, practical implementations of RBPF-based SLAM algorithms rely on the parallelization of the particle filter, which permits an increased number of particles. Therefore, unlike in multi-target tracking, the LMB filter may lose its ability to perform efficient map updates within a parallelized RBPF implementation of the LMB-SLAM algorithm.

All SLAM algorithms that adopt standard RBPF-based robot trajectory estimation methods (including PHD-SLAM and LMB-SLAM) suffer from particle degeneration due to resampling and mismatch between the proposal and target distributions. This happens when proposal distributions designed to use only robot controls (which are often less certain and modeled with a large covariance), sample particles in a large area of the state space. As a result, after the measurement update, only those particles, which have maps that closely match the measurements, receive high weights. Therefore, the vast majority of particles receive negligible weights. During resampling, the higher weighted particles are duplicated, thus eliminating the particles with lower weights resulting in loss of particle diversity. This reduces the filter’s ability to revise the path of the robot and results in divergence over time. To address this issue, Montemerlo et al. proposed the FastSLAM 2.0 algorithm, which incorporates the current observations and the existing map to further improve the proposal distribution to closely match the robot trajectory posterior [23,24].

In this article, an RFS-based SLAM algorithm called δ-GLMB-SLAM2.0 is proposed using the optimal kernel-based RBPF approach [25] for robot trajectory estimation, and the recently developed efficient δ-GLMB filter [15] for landmark map estimation. Preliminary results of the proposed algorithm hence referred to as δ-GLMB-SLAM1.0, were published in [26]. An improved version is presented here, referred to as δ-GLMB-SLAM2.0, where the RBPF used in the trajectory estimation adopts a modified proposal distribution similar to FastSLAM 2.0, which results in superior performance. Furthermore, δ-GLMB-SLAM2.0 benefits from substantial computational savings in landmark map estimation due to inheriting the Gibbs sampler-based joint prediction/update method of the efficient δ-GLMB filter [15]. It is demonstrated that the proposed δ-GLMB-SLAM2.0 algorithm outperforms the original δ-GLMB-SLAM1.0 [26] and LMB-SLAM algorithms in terms of pose estimation error and quality of the map, and yields superior, robust performance under feature detection uncertainty and varying clutter rates, while only slightly compromising the computational cost.

## 2. Problem Formulation

Let $u_{1:k}={\left[u_{1},u_{2},\ldots ,u_{k}\right]}^{T}$ denote the time sequence of control commands applied to the robot up to time *k*, where $u_{k}$ denotes the control command applied at time *k*. In addition, let the time sequence of the pose history of the robot be denoted by $x_{1:k}={\left[x_{1},x_{2},\ldots ,x_{k}\right]}^{T}$, where $x_{k}$ denotes the pose of the robot with respect to the global frame of reference at time *k*. Further, let the time sequence of sets of measurements be denoted by $Z_{1:k}=\left[Z_{1},Z_{2},\ldots ,Z_{k}\right]$, where $Z_{k}=\left\{z_{k,1},z_{k,2},\ldots ,z_{k,m_{k}}\right\}$ denotes the measurement set received at time *k*, and $m_{k}$ denotes the number of detections. These measurements result from detections collected from an exteroceptive sensor mounted on the robot, such as a camera, lidar or a sonar, using a feature extraction algorithm.

### 2.1. Labeled RFS Representation of the Map

Let the landmark map be represented as a labeled RFS $M=\{{\tilde{m}}_{k,1},{\tilde{m}}_{k,2},\ldots ,{\tilde{m}}_{k,n_{k}}\}$ where $n_{k}$ denotes the number of landmarks present in the environment at time *k*. Each realization of a landmark $\tilde{m}\in M$ is of the form $\tilde{m}=\left(m,l\right)$, where m∈M is the kinematic state and l∈L is a distinct label of the point **m**. Distinct labels provide a method to distinguish between landmarks [12,13].

Let the kinematic state space of a landmark be denoted by M and the discrete label space be denoted by L. Further, let L:M×L→L be the projection from labeled RFSs to labels defined by L(m,l)=l. Let the Kronecker delta function for arbitrary arguments (such as vectors, sets or integers) be denoted by $\delta_{Y}\left(X\right)$, which takes the value of 1 only if X=Y and 0 otherwise. The indicator function $1_{X}\left(Y\right)$ takes the value of 1 if Y∈X and 0 otherwise. Let $\Delta \left(M\right)\overset{\Delta }{\;=}\delta_{\left|M\right|}\left(\left|L(M)\right|\right)$ denote the distinct label indicator, which takes the value of 1 if and only if the labeled set M has the same cardinality as its labels $L\left(M\right)=\{L\left(\tilde{m}\right):\tilde{m}\in M\}$ and 0 otherwise. Let F(J) represent all finite subsets of a set J. The inner product of two continuous functions f(x) and g(x) is denoted by $\langle f,g\rangle \overset{\Delta }{=}\int f(x)g(x)dx$ and for a real valued function h(x), the multi-object exponential is defined as $h{\left(\cdot \right)}^{X}≜\prod_{x\in X}h(x)$.

### 2.2. Rao–Blackwellization of the SLAM Problem

In the SLAM problem, it is necessary to evaluate the posterior probability distribution,

(1) $$p_{k|k}\left(M_{k},x_{1:k}|Z_{1:k},u_{1:k},x_{0}\right)$$

for all times *k*, where $x_{0}$ denotes the initial pose of the vehicle. In other words, it is necessary to evaluate the joint posterior density consisting of the map and the robot pose history at all times *k*, given the time sequences of sets of observations, and control commands up to and including time *k* and the initial robot pose.

The joint posterior density consisting of the landmark map $M_{k}$ and robot trajectory $x_{1:k}$ at time *k*, is evaluated using the existing map $M_{k-1}$, history of robot poses $x_{0:k-1}$ evaluated at time k−1, the time sequence of sets of observations $Z_{1:k}$ and the applied control commands $u_{1:k}$ up to and including time *k*. In a manner similar to Montemerlo’s approach in FastSLAM [3], the SLAM posterior is factorized into a product of the robot trajectory posterior and the map posterior conditioned on the robot trajectory as follows,

(2) $$\begin{array}{c} p_{k|k}\left(M_{k},x_{1:k}|Z_{1:k},u_{1:k},x_{0}\right)=p_{k|k}\left(M_{k}|Z_{1:k},x_{0:k}\right)\times p_{k|k}\left(x_{1:k}|Z_{1:k},u_{1:k},x_{0}\right). \end{array}$$

This decouples the SLAM problem into two separate (conditionally independent) estimation problems. The key advantage of this factorization is two-fold: Firstly it can benefit from Monte Carlo methods (particle filtering) for joint robot trajectory estimation, making it possible to adopt non-linear motion models. Secondly, it can benefit from using an RFS representation and Finite Set Statistic (FISST) techniques for landmark map posterior estimation. By representing the map and measurements as RFSs and appropriately modeling the map transition model, it is possible to estimate the number and locations of multiple landmarks in the presence of measurement noise and clutter within a single joint estimation framework. This automatically considers all data association hypotheses and takes landmark detection and survival uncertainties into account.

### 2.3. δ-GLMB-SLAM Observation Model

The measurement set received from the sensor at time *k* contains both landmark generated measurements and false alarms (measurement clutter). Let the RFS $C_{k}$ denote the measurement clutter. Then, the measurements received at time *k* can be modeled by the RFS,

(3) $$Z_{k}=C_{k}\cup \left[\underset{\left(m_{k},l_{k}\right)\in M_{k}}{⋃}H_{k}\left(m_{k},l_{k}\right)\right]$$

where $H_{k}\left(m_{k},l_{k}\right)$ is a Bernoulli RFS representing the measurement corresponding to the observation of landmark $\left(m_{k},l_{k}\right)\in M_{k}$. Due to the limited field of view (FOV) of the sensor, $H_{k}\left(m_{k},l_{k}\right)$ can have a value of the form $\left\{z_{k}\right\}$ with probability of detection $p_{D}\left(m_{k},l_{k}|x_{k}\right)$ or *∅* with probability of $1-p_{D}\left(m_{k},l_{k}|x_{k}\right)$. Note that the probability of detection is a function of the landmark state and robot position. The measurement likelihood function, conditioned on the detection of the landmark with state $\left(m_{k},l_{k}\right)$, is given by $g_{k}\left(z_{k}|m_{k},l_{k},x_{k}\right)$. Assuming that the measurements are conditionally independent and the measurement clutter is an independent process, the measurement likelihood function corresponding to the observations can be written using Equations (12.139) and (12.140) of Chapter 12 in [9] as,

(4) $$g\left(Z|M,x\right)=e^{-\langle \kappa ,1\rangle }\kappa^{Z}\sum_{\theta \in \Theta (L(M))}{\left[\psi_{Z}\left(\cdot ;\theta \right)\right]}^{M},$$

where κ denotes the intensity of Poisson distributed measurement clutter, and

(5) $$\psi_{Z}\left(m,l;\theta \right)=\left\{\begin{array}{cc} \frac{p_{D}\left(m,l\right)g\left(z_{\theta (l)}|m,l,x\right)}{\kappa (z_{\theta (l)})} & \mathrm{if}\;\theta (l)>0\\ 1-p_{D}\left(m,l\right) & \mathrm{if}\;\theta (l)=0, \end{array}\right.$$

where θ is an association map of the form, θ:L→0,1,…,|Z| such that each and every distinct estimated feature is associated to a distinct measurement (i.e., $\theta \left(i\right)=\theta \left(i^{\prime }\right)>0$ implies $i=i^{\prime }$). The set Θ of all such association maps is called the association map space and a subset of association maps with domain I is denoted by Θ(I). Note that unlike multi-target tracking, in SLAM, previously estimated landmarks that exit the current sensor FOV are retained in the map with probability of detection $p_{D}\left(m,l\right)=0$ during the measurement update step and therefore in general contribute to the robot trajectory posterior estimate.

### 2.4. δ-GLMB-SLAM Map Transition Model

As the robot continues to explore the unknown environment, new observations are collected in the limited FOV of the sensor and fused into the landmark map. These new landmarks are modeled as labeled RFS $Q_{k}$ with the birth label space B, and the corresponding birth density is assumed to be a labeled multi-Bernoulli density and can be modeled in a similar manner to Equation (2) from [15] as,

(6) $$f_{B}\left(Q_{k}\right)=\Delta \left(Q\right){\left[1-r_{B}^{\left(.\right)}\right]}^{B-L(Q_{k})}{\left[1_{B}\left(.\right)r_{B}^{\left(.\right)}\right]}^{L(Q_{k})}{\left[p_{B}\right]}^{Q_{k}},$$

where a realization of $r_{B}^{\left(\cdot \right)}$ is of the form $r_{B}^{\left(l\right)}=r_{B}\left(m,l\right)$ for any label l∈B and denotes the birth probability of the landmark with label *l* and $p_{B}\left(m,l\right)$ denotes its spatial distribution.

Furthermore, a portion of the already existing landmarks in the map appears in the current sensor FOV. Given the state of the current landmark map, M, a landmark $\left(m_{k},l_{k}\right)\in M$ may appear in the sensor FOV in the next time step with survival probability $p_{S}\left(m_{k},l_{k}|x_{k}\right)$ and changes its state to $\left(m_{k+1},l_{k+1}\right)$ with probability density $\delta_{m_{k}}\left(m_{k+1}\right)\delta_{l_{k}}\left(l_{k+1}\right)p\left(m_{k},l_{k}\right)$, or leave the sensor FOV with probability $q_{S}\left(m_{k},l_{k}|x_{k}\right)=1-p_{S}\left(m_{k},l_{k}|x_{k}\right)$. It is also important to note that, unlike multi-target tracking, in SLAM, landmarks are usually assumed stationary and hence the motion of a landmark is modeled as a Kronecker delta function, $\delta_{m_{k}}\left(m_{k+1}\right)$. In addition, the label of a landmark is preserved during the state transition. Assuming that the state of the landmark map is represented by M, the set of surviving landmarks in the next time step is modeled as a labeled multi-Bernoulli (LMB) RFS W with parameter set $\left\{\left(p_{S}\left(m,l|x\right),\delta_{m}\left(\cdot \right)\delta_{l}\left(\cdot \right)p\left(m,l\right)\right):\left(m,l\right)\in M\right\}$. Then, the state transition of the map can be modeled using a LMB distribution similar to Equation (25) from [12] as,

(7) $$f_{S}\left(W|M\right)=\Delta \left(W\right)\Delta \left(M\right)1_{L(M)}\left(L\left(W\right)\right){\left[\Phi \left(W;\cdot \right)\right]}^{M},$$

where

(8) $$\begin{array}{cc} \Phi (W;m_{k+1},l_{k+1}) & =\sum_{(m_{k+1},l_{k+1})\in W}\;\;\;\;\;\;\;\;\;\;\;p_{S}\left(m_{k},l_{k}|x_{k}\right)\delta_{m_{k}}\left(m_{k+1}\right)\delta_{l_{k}}\left(l_{k+1}\right)p\left(m_{k},l_{k}\right)+\left[1-1_{L(W)}\left(l_{k}\right)\right]q_{S}\left(m_{k},l_{k}|x_{k}\right). \end{array}$$

The first part in Equation (8) corresponds to the surviving landmarks and the second corresponds to dying (disappearing) landmarks. The newly appearing (birth) landmarks are independent of the already existing landmarks in the map. Therefore, the probability density of the predicted state of the map $M_{k+1}$, conditioned on the current map $M_{k}$, can be written as a product of birth density and the transition density of the surviving landmarks similar to Equation (31) from [12] as follows,

(9) $$\begin{array}{cc} f(M_{k+1}|M_{k}) & =f_{S}\left(M_{k+1}\cap \left(M\times L\right)|M_{k}\right)\times f_{B}\left(M_{k+1}-\left(M\times L\right)\right). \end{array}$$

Note that the estimated landmarks that exit the current sensor FOV should remain in the map and be modeled with probability of survival $p_{S}\left(m,l|x\right)=1$ during the prediction step and contribute to the robot trajectory posterior estimate.

### 2.5. δ-GLMB-SLAM Map Estimation

To propagate the landmark map in time, we adopt the recently proposed efficient δ-GLMB filter [15]. This approach avoids the traditional prediction/update steps of a Bayesian filter and directly updates the posterior at time *k* to time k+1, achieving significant computational savings compared to its original implementation [12,13]. Let the map posterior $p(M_{k}|Z_{1:k},x_{0:k})$ at time *k* be abbreviated as p(M); let the measurement updated landmark map posterior at time k+1 be abbreviated as $p(M_{+}|Z_{+})$; and let the robot pose at time k+1 be denoted by $x_{+}$. Assume that p(M) at time *k* is given by the δ-GLMB distribution of the following form [12],

(10) $$\begin{array}{cc} p(M)=\Delta (M) & \;\;\;\;\;\;\;\;\;\;\sum_{(I,\xi )\in F(L)\times \Xi }\omega^{\left(I,\xi \right)}\delta_{I}\left(L\left(M\right)\right){\left[p^{\left(\xi \right)}\right]}^{M}, \end{array}$$

where I∈F(L) represents a set of landmark labels and ξ∈Ξ represents a history of association maps up to time *k* and denoted by $\xi =(\theta_{1},\ldots ,\theta_{k})$. The pair (I,ξ) represents the hypothesis that the set of landmarks with labels I has history ξ of association maps. The weight $\omega^{\left(I,\xi \right)}$ represents the probability of the hypothesis (I,ξ), and $p^{\left(\xi \right)}\left(m,l\right)$ represents the probability density of the kinematic state of the feature with label *l* and the association map history ξ.

Assume that the birth landmarks (newly appearing landmarks) and the surviving landmarks in the FOV follow LMB distributions as in Equations (6) and (7). Let $B_{+}$ denote the label space of newly appearing features in the FOV at time k+1. Then, adopting the joint prediction/update approach proposed in [15], the measurement updated map posterior $p(M_{+}|Z_{+})$ can be written as,

(11) $$\begin{array}{cc} p(M_{+}|Z_{+}) & \propto \Delta \left(M_{+}\right)\sum_{I,\xi ,I_{+},\theta_{+}}\omega^{\left(I,\xi \right)}\omega_{Z_{+}}^{\left(I,\xi ,I_{+},\theta_{+}\right)}\delta_{I_{+}}\left(L\left(M_{+}\right)\right){\left[p_{Z_{+}}^{\left(\xi ,\theta_{+}\right)}\right]}^{M_{+}}, \end{array}$$

where $I_{+}\in F\left(L_{+}\right)$ and $\theta_{+}\in \Theta_{+}$, $L_{+}=L_{}\cup B_{+}$ and $\Theta_{+}$ denotes the association map space at time k+1, and

(12) $$\begin{array}{cc} w_{Z_{+}}^{\left(I,\xi ,I_{+},\theta_{+}\right)} & =1_{\Theta_{+}\left(I_{+}\right)}\left(\theta_{}+\right){\left[1-{\bar{P}}_{S}^{\left(\xi \right)}\right]}^{I-I_{+}}{\left[{\bar{P}}_{S}\right]}^{I\cap I_{+}}{\left[1-r_{B,+}^{\left(\cdot \right)}\right]}^{\left(B_{+}-I_{+}\right)}r_{B,+}^{\left(B_{+}\cap I_{+}\right)}{\left[{\bar{\psi }}_{Z_{+}}^{\left(\xi ,\theta_{+}\right)}\right]}^{I_{+}}, \end{array}$$

(13) $${\bar{P}}_{S}^{\left(\xi \right)}\left(l\right)=\langle p^{\left(\xi \right)}\left(\cdot ,l\right),p_{S}\left(\cdot ,l|x_{+}\right)\rangle ,$$

(14) $${\bar{\psi }}_{Z_{+}}^{\left(\xi ,\theta_{+}\right)}\left(l_{+}\right)=\langle {\bar{p}}_{+}^{\left(\xi \right)}\left(\cdot ,l_{+}\right),\psi_{Z_{+}}^{\left(\theta_{+}\left(l_{+}\right)\right)}\left(\cdot ,l_{+}\right)\rangle ,$$

where $\psi_{Z_{+}}^{\left(\theta_{+}\left(l_{+}\right)\right)}\left(m_{+},l_{+}\right)=\psi_{Z_{+}}\left(m_{+},l_{+};\theta_{+}\right)$ (see Equation (5)), and

(15) $$\begin{array}{cc} {\bar{p}}_{+}^{\left(\xi \right)}\left(m_{+},l_{+}\right) & =1_{L}\left(l_{+}\right)\frac{\langle p_{S}\left(\cdot ,l_{+}|x_{+}\right)\delta_{\left(\cdot \right)}\left(m_{+}\right)\delta_{\left(\cdot \right)}\left(l_{+}\right),p^{\left(\xi \right)}\left(\cdot ,l_{+}\right)\rangle }{{\bar{P}}_{S}^{\left(\xi \right)}\left(l_{+}\right)}+1_{B_{+}}\left(l_{+}\right)p_{B,+}\left(m_{+},l_{+}\right), \end{array}$$

(16) $$p_{Z_{+}}^{\left(\xi ,\theta_{+}\right)}\left(m_{+},l_{+}\right)=\frac{{\bar{p}}_{+}^{\left(\xi \right)}\left(m_{+},l_{+}\right)\psi_{Z_{+}}^{\left(\theta_{+}\left(l_{+}\right)\right)}\left(m_{+},l_{+}\right)}{{\bar{\psi }}_{Z_{+}}^{\left(\xi ,\theta_{+}\right)}\left(l_{+}\right)},$$

where the notation + has been used to abbreviate the symbols at time k+1 and $r_{B,+}^{\left(l_{+}\right)}$ denotes the probability of birth of the landmark with label $l_{+}$. Note that the resultant map update in Equation (11) is a δ-GLMB distribution, which is equivalent in its form to the prior distribution in Equation (10). Each prior map hypothesis (I,ξ) generates a set of new hypotheses $\left(I,\xi ,I_{+},\theta_{+}\right)$ using the robot position, prior spatial distributions, birth spatial distributions, probability of detection and probability of survival values of landmarks, probability of births and the set of received measurements at time k+1. Equations (12)–(14) contribute to the hypotheses weights and result in scalar values. Equation (15) is the spatial distribution of a predicted landmark, where the first part corresponds to a survival and the second to a birth. Equation (16) corresponds to the spatial distribution after measurement update. Note that the measurement update step takes measurement to track associations into account to generate the resultant spatial distribution.

The idea behind the joint prediction/update approach is to generate a smaller number of highly probable map hypotheses using existing hypotheses at time *k*, by simulating most likely data association decisions, detection/miss-detection decisions and survival/death possibilities using a Gibbs sampler. This prevents the generation of insignificant and contradicting hypotheses and drastically reduces the computational complexity compared to the traditional prediction/update based δ-GLMB filter implementation [13]. It also yields a computationally efficient alternative for real-time implementations.

We assume that the probability of detection $p_{D}\left(m,l|x\right)$, and probability of survival $p_{S}\left(m,l|x\right)$ have constant values depending on whether a landmark is within the sensor FOV or not, and the measurement likelihood is modeled as a Gaussian probability density function. Furthermore, the spatial distribution of each birth landmark, $p_{B,+}\left(m_{+},l_{+}\right)$, is modeled as a Gaussian (or mixture of Gaussians) probability density function. Hence, the δ-GLMB filter follows a Gaussian mixture representation, where the spatial distribution of each landmark in the map with label *l*, and an association map history ξ in each hypothesis, results in a Gaussian (or a mixture of Gaussians) probability density function.

### 2.6. Trajectory Estimation

In a manner similar to PHD-SLAM [18] (and LMB-SLAM [20]), the robot trajectory posterior is factorized and further simplified using the Markov property as follows,

(17) $$\begin{array}{cc} p_{k|k}\left(x_{1:k}|Z_{1:k},u_{1:k},x_{0}\right) & =\frac{g_{k|k-1}\left(Z_{k}|Z_{k-1},x_{0:k}\right)p_{k|k-1}\left(x_{k}|x_{k-1},u_{k}\right)}{p(Z_{k}|Z_{k-1},u_{1:k},x_{0})}\\ & \times p_{k-1|k-1}\left(x_{1:k-1}|Z_{1:k-1},u_{1:k-1},x_{0}\right), \end{array}$$

and to cater for non-linear and non-Gaussian motion models, a Rao–Blackwellized particle filter [27] is adopted, with an improved sampling distribution motivated by the FastSLAM 2.0 algorithm [23]. Similar to FastSLAM 2.0, the proposal distribution is chosen to include the current observation set so that sampling not only takes robot controls into account, but also the current measurement set. Let the proposal distribution be $q(x_{1:k}|Z_{1:k},u_{1:k},x_{0})$, which can be factorized and simplified using the Markov property as,

(18) $$q_{k|k}\left(x_{1:k}|Z_{1:k},u_{1:k},x_{0}\right)=q\left(x_{k}|x_{0:k-1},Z_{1:k},u_{1:k}\right)\times q_{k-1|k-1}\left(x_{1:k-1}|Z_{1:k-1},u_{1:k-1},x_{0}\right),$$

where the sampling function can be written as,

(19) $$\begin{array}{cc} q(x|x_{0:k-1},Z_{1:k},u_{1:k}) & =\eta \int g\left(Z_{k}|M,x\right)p_{k|k-1}\left(M|Z_{1:k-1},x,x_{0:k-1}\right)\delta M\\ & \times q(x|x_{k-1},u_{k}), \end{array}$$

where η is a normalization constant.

Note that although Equations (17) and (18) appear to be similar, the proposed distribution in Equation (18) is chosen to have a form which can be efficiently sampled, while the target distribution in Equation (17) is, in general, more difficult to sample.

Note that the denominator of the target distribution, $p(Z_{k}|Z_{k-1},u_{1:k},x_{0})$, is a normalization constant. The state transition function of the robot trajectory distribution is known and the state transition function of the proposal distribution is chosen to be equivalent to that of the robot trajectory posterior such that,

(20) $$\begin{array}{c} q_{k|k-1}\left(x_{k}|x_{k-1},u_{k}\right)=p_{k|k-1}\left(x_{k}|x_{k-1},u_{k}\right). \end{array}$$

Then, sampling a robot pose $x_{k}$ from the proposal distribution is equivalent to sampling from the transition density given by,

(21) $$x_{k}∼\left[\int g\left(Z_{k}|M,x\right)p_{k|k-1}\left(M|Z_{1:k-1},x,x_{0:k-1}\right)\delta M\right]q_{k|k-1}\left(x|x_{k-1},u_{k}\right),$$

and the weighting function can be evaluated as follows,

(22) $$\begin{array}{cc} w_{k} & =\frac{p(x_{1:k}|Z_{1:k},u_{1:k},x_{0})}{q(x_{1:k}|Z_{1:k},u_{1:k},x_{0})}\\ & \propto \int \int g\left(Z_{k}|M,x\right)p_{k|k-1}\left(M|Z_{1:k-1},x,x_{0:k-1}\right)q_{k|k-1}\left(x|x_{k-1},u_{k}\right)\delta Mdx\times w_{k-1}. \end{array}$$

It is important to note that, to sample a robot pose $x_{k}$ in Equation (21), the map posterior at time k−1 is used during the sampling step and the same integral is evaluated again at the weight update step in Equation (22) with the sampled robot pose.

## 3. Implementation

This section presents the implementation details of the proposed δ-GLMB-SLAM algorithm. The robot trajectory is propagated using a Rao–Blackwellized particle filter to cater for non-linear and possibly multi-modal motion models in both 2D and 3D environments. The trajectory dependant landmark map is modeled as a labeled RFS and propagated using a δ-GLMB filter [15].

The δ-GLMB distribution of the landmark map posterior at time *k* can be approximated using a set of *H* highest probable hypotheses in the following form,

(23) $$p\left(M\right)=\Delta \left(M\right)\sum_{h=1}^{H}{\omega^{\left(h\right)}\delta_{I^{\left(h\right)}}\left(L\left(M\right)\right)\left[p^{\left(h\right)}\right]}^{M},$$

where the right hand side of the above equation can also be represented as a parameter set of the form ${\left\{(I^{\left(h\right)},\omega^{\left(h\right)},p^{\left(h\right)})\right\}}_{h=1}^{H}$, where, for each hypothesis *h*, $I^{\left(h\right)}$ represents a set of landmark labels, $\omega^{\left(h\right)}$ represents the probability of the hypothesis and $p^{\left(h\right)}$ consists of the spatial distribution $p^{\left(h\right)}\left(m,l\right)$ of each landmark within this hypothesis.

Suppose that the robot trajectory posterior, $p_{k|k}\left(x_{1:k}|Z_{1:k},u_{1:k},x_{0}\right)$ can be represented by a set of weighted particles of the form $\Omega_{k}={\left\{w_{k}^{\left[i\right]},x_{0:k}^{\left[i\right]}\right\}}_{i=1}^{N_{s}}$, where $w^{\left[i\right]}$ represents the weight of the particle *i*. Then, the SLAM posterior (Equation (1) can be represented as,

(24) $${\left\{w_{k}^{\left[i\right]},x_{0:k}^{\left[i\right]},{\left\{(I^{\left(i,h\right)},\omega^{\left(i,h\right)},p^{\left(i,h\right)})\right\}}_{h=1}^{H}\right\}}_{i=1}^{N_{s}},$$

since the δ-GLMB distribution of the landmark map is conditioned on the robot trajectory. The details of the implementation of the particle filter and the Gaussian mixture (GM) implementation of the δ-GLMB filter is given in the following subsections.

### 3.1. Map Estimation

In this section, we briefly summarize the details of the Gaussian mixture implementation of the δ-GLMB filter used in the estimation of the landmark map. Let N(·,μ,P) denote a Gaussian probability density function with mean μ and covariance P, and assume that the probability of detection of a landmark within the sensor FOV is of the form $p_{D}=p_{D}\left(m,l|x\right)$. Let the observation model be a non-linear function of the form,

(25) $$z_{k}=h_{k}\left(m_{k},l_{k},x_{k}^{\left[i\right]},\nu_{k}\right),$$

where $\nu_{k}$ represents a zero-mean Gaussian measurement noise source with covariance $R_{k}$ and ${x_{k}}^{\left[i\right]}$ is the robot pose according to particle *i* at time *k*. Then, assuming that the spatial distribution of $\left(m_{k},l_{k}\right)$ is of the form $N(m_{k};\mu_{k},P_{k})$, the measurement likelihood can be approximated as a Gaussian distribution by linearizing as follows:

(26) $$\begin{array}{c} g_{k}\left(z|m_{k},l_{k},x_{k}^{\left[i\right]}\right)\approx N\left(z;h_{k}\left(\mu_{k},l_{k},x_{k}^{\left[i\right]},0\right),U_{k}R_{k}{U_{k}}^{T}+H_{k}P_{k}{H_{k}}^{T}\right), \end{array}$$

where $U_{k}$ is the Jacobian of $h_{k}\left(m_{k},l_{k},x_{k}^{\left[i\right]},\nu_{k}\right)$ with respect to $\nu_{k}$ at $\nu_{k}=0$ (0 is the zero vector) and $H_{k}$ denotes the Jacobian of $h_{k}\left(m_{k},l_{k},x_{k}^{\left[i\right]},0\right)$ with respect to $m_{k},$ at $m_{k}=\mu_{k}$.

Furthermore, assume that the RFS $Q_{+}$ of newly appearing features in the sensor FOV can be modeled by a labeled multi-Bernoulli (LMB) distribution given by,

(27) $$f_{B}\left(Q_{+}\right)={\left\{r_{B,+}^{\left(l_{+}\right)},p_{B,+}\left(m_{+},l_{+}|z_{+}\right)\right\}}_{l_{+}=1}^{\left|Z_{+}\right|},$$

where $r_{B,+}^{\left(l_{+}\right)}$ denotes the probability of birth of landmark $l_{+}$, and its spatial distribution $p_{B,+}\left(m_{+},l_{+}|z_{+}\right)$ is modeled as a Gaussian distribution (or a mixture of Gaussian distributions) using the adaptive birth approach proposed in [14]. Assume that each hypothesis of the landmark map distribution p(M) (Equation (10)) at time *k* is represented by the hypothesis weight $\omega^{\left(I,\xi \right)}$. In addition, assume that the set of spatial distributions of each landmark in the set I with label *l* is given by a mixture of weighted Gaussian distributions as,

(28) $$p^{\left(\xi \right)}\left(m,l\right)=\sum_{j=1}^{J^{\left(\xi \right)}\left(l\right)}\alpha_{j}^{\left(\xi \right)}N\left(m;\mu_{j}^{\left(\xi \right)}\left(l\right),P_{j}^{\left(\xi \right)}\left(l\right)\right),$$

where $\alpha_{j}^{\left(\xi \right)}$ denotes the weight of *j*th Gaussian component. Then, using Equations (14)–(16) it can be shown that the measurement updated spatial density for a given association map $\theta_{+}$ in the measurement updated δ-GLMB distribution (Equation (11)) also results in a mixture of weighted Gaussian distributions of the form,

(29) $$p_{Z_{+}}^{\left(\xi ,\theta_{+}\right)}\left(m,l\right)=\sum_{j=1}^{J^{\left(\xi \right)}\left(l\right)}\alpha_{Z_{+},j}^{\left(\xi ,\theta_{+}\right)}N\left(m;\mu_{Z_{+},j}^{\left(\xi ,\theta_{+}\right)}\left(l\right),P_{Z_{+},j}^{\left(\xi ,\theta_{+}\right)}\left(l\right)\right),$$

where $\alpha_{Z_{+},j}^{\left(\xi ,\theta_{+}\right)}$, $\mu_{Z_{+},j}^{\left(\xi ,\theta_{+}\right)}\left(l\right)$ and $P_{Z_{+},j}^{\left(\xi ,\theta_{+}\right)}\left(l\right)$ denote the weight, mean and covariance of the measurement updated *j*th Gaussian distribution component using the association map $\theta_{+}$, respectively. The weight ${\bar{\omega }}_{Z_{+}}^{\left(I,\xi ,I_{+},\theta_{+}\right)}$ of each hypothesis can be obtained using Equations (12)–(15). Note that the sum of the hypothesis weights, is equivalent to the normalization constant of the δ-GLMB update posterior (Equation (11)), and is used in the trajectory update step in Section 3.2.

To extract the landmark map, the cardinality distribution component of the δ-GLMB distribution of the landmark map of the highest weighted particle is obtained using its hypothesis weights. The highest weighted hypothesis component with the cardinality equivalent to the maximum a posteriori (MAP) cardinality estimate (see [13]) is assumed to contain the labels and the mean locations of the landmarks in the map.

### 3.2. Robot Trajectory Estimation

Assume that the weighted set of particles $\Omega_{k-1}$ represents the robot trajectory posterior at time k−1. Then, at time *k*, a new robot pose is sampled from each particle using controls, measurements and the map posterior at time k−1 as follows,

(30) $$x_{k}^{\left[i\right]}∼\left[\int g\left(Z_{k}|M,x\right)p_{k|k-1}\left(M|Z_{1:k-1},x,x_{0:k-1}^{\left[i\right]}\right)\delta M\right]q_{k|k-1}\left(x|x_{k-1}^{\left[i\right]},u_{k}\right),$$

where the robot pose transition due to a control input alone is modeled using a non-linear function given by,

(31) $$x=f_{x}\left(x_{k-1},u_{k},\epsilon_{k}\right),$$

where $\epsilon_{k}$ denotes Gaussian white control noise with covariance $Q_{k}$. The robot pose transition function (due to control inputs) is modeled as a Gaussian probability density function given by,

(32) $$q_{k|k-1}\left(x|x_{k-1}^{\left[i\right]},u_{k}\right)=N\left(x|f_{x}\left(x_{k-1}^{\left[i\right]},u_{k},0\right),F_{x,k-1}^{\left[i\right]}P_{x,k-1}^{\left[i\right]}{F^{\left[i\right]}}_{x,k-1}^{T}+F_{\epsilon ,k}Q_{k}F_{\epsilon ,k}^{T}\right).$$

In Equation (32), $F_{\epsilon ,k}$ denotes the Jacobian of the robot motion model (Equation (31)) with respect to the control noise when the control noise $\epsilon_{k}=0$. Further, $F_{x,k-1}^{\left[i\right]}$ denotes the Jacobian of Equation (31) with respect to robot pose at $x_{k-1}^{\left[i\right]}$, and $P_{x,k-1}^{\left[i\right]}$ denotes the pose covariance. Note that we adopt the Gaussian mixture implementation of the δ-GLMB filter, thus the right hand side of Equation (30) results in a single Gaussian probability density function, which is a summation of a set of weighted Gaussian probability density functions, from which sample $x_{k}^{\left[i\right]}$ is drawn.

The new robot pose, $x_{k}^{\left[i\right]}$, is then added to the set of particles $\Omega_{k-1}$, creating a temporary set of particles, all of which have been drawn from the proposal distribution. Now, each particle in the temporary set is assigned an importance weight given by,

(33) $$\begin{array}{c} w_{k}^{\left[i\right]}\propto \int \int g\left(Z_{k}|M,x\right)p_{k|k-1}\left(M|Z_{1:k-1},x,x_{0:k-1}\right)q_{k|k-1}\left(x|x_{k-1},u_{k}\right)\delta Mdx\times w_{k-1}, \end{array}$$

The importance weight of each particle in the temporary set is normalized such that $\sum_{i=1}^{N_{s}}w_{k}^{\left[i\right]}=1$. Then, a new set of $N_{s}$ particles is drawn with replacement, where each particle is sampled with a probability proportional to its importance weight. The resultant particle set with its importance weights, denoted by $\Omega_{k}$, represents the robot trajectory posterior density at time *k*.

## 4. Results

### 4.1. Simulated Results

The performance of the proposed δ-GLMB-SLAM algorithm (hereafter referred to as δ-GLMB-SLAM2.0) was evaluated using a set of Matlab simulations and compared with the recent δ-GLMB-SLAM algorithm [26] (hereafter referred to as δ-GLMB-SLAM1.0) and an efficient variant of LMB-SLAM using the recently proposed fast implementation of the LMB filter with a Gibbs sampler-based efficient state hypothesis generation approach [28]. The Rao–Blackwellized particle filters in LMB-SLAM, δ-GLMB-SLAM1.0 and δ-GLMB-SLAM2.0 were implemented using the Matlab parallel computing toolbox. Standard measurement gating approaches were used in all three algorithms with identical parameters to reduce the computational costs. However, parallelization of the particle filter prevented the execution of parallel measurement updates in the LMB filter, thus measurement clustering in the LMB-SLAM implementation was not used.

A robot was driven on a pre-planned path in a simulated environment consisting of 23 landmarks. The control commands and measurements were generated from a single run of the robot with added Gaussian noise according to the parameters in Table 1 and measurement clutter was generated in four separate runs with rates $\lambda_{c}=$ 1, 5, 10 and 15 points per scan. The probability of detection $p_{D}$ of a landmark within the sensor FOV was set to 0.7 and the detection probability $p_{D}$ of a landmark already existing in the map, but out of the FOV, was set to 0. The probability of survival $p_{S}$ of a feature within the sensor FOV was set to 0.95 and that of a landmark already existing in the map and out of the current sensor FOV was set to 1. These settings ensured that a landmark in the estimated map remained in the map when it left the current sensor FOV, which was consistent with the assumption that the landmarks remained static. Newly appearing birth features were modeled using the adaptive birth approach [20] and a birth probability $r_{B,+}^{\left(l_{+}\right)}$ value of 0.0001 was used for new features and a $r_{B,+}^{\left(l_{+}\right)}$ value of 0.00005 was used for existing ones. These birth probability values were chosen to prevent newly appearing landmarks from replacing already existing ones in the current sensor FOV. A pruning threshold of 0.019 was chosen for the probability of existence in LMB-SLAM to prune insignificant features created due to measurement clutter. A hypothesis pruning threshold of 0.00001 was chosen in both δ-GLMB-SLAM1.0 and δ-GLMB-SLAM2.0 to remove insignificant hypotheses, which resulted in a reduced computational cost during map update. These values produced comparable estimation results and were chosen by executing the simulations (without control noise and measurement clutter) multiple times with LMB-SLAM, δ-GLMB-SLAM1.0 and δ-GLMB-SLAM2.0 under all four clutter conditions.

**Figure 1:**
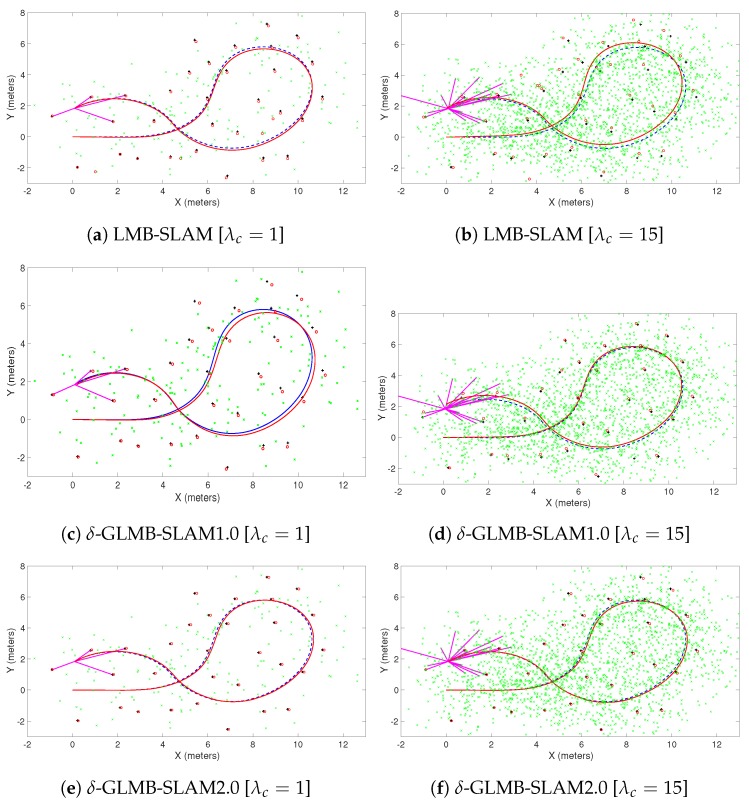
A comparison of the estimated robot trajectory (red) superimposed on the ground truth robot trajectory (dashed blue) for varying clutter conditions. The black plus signs represent the actual feature positions and the red circles represent the estimated feature positions. The green crosses represent accumulated measurement clutter and the magenta lines correspond to feature observations.

**Figure 2:**
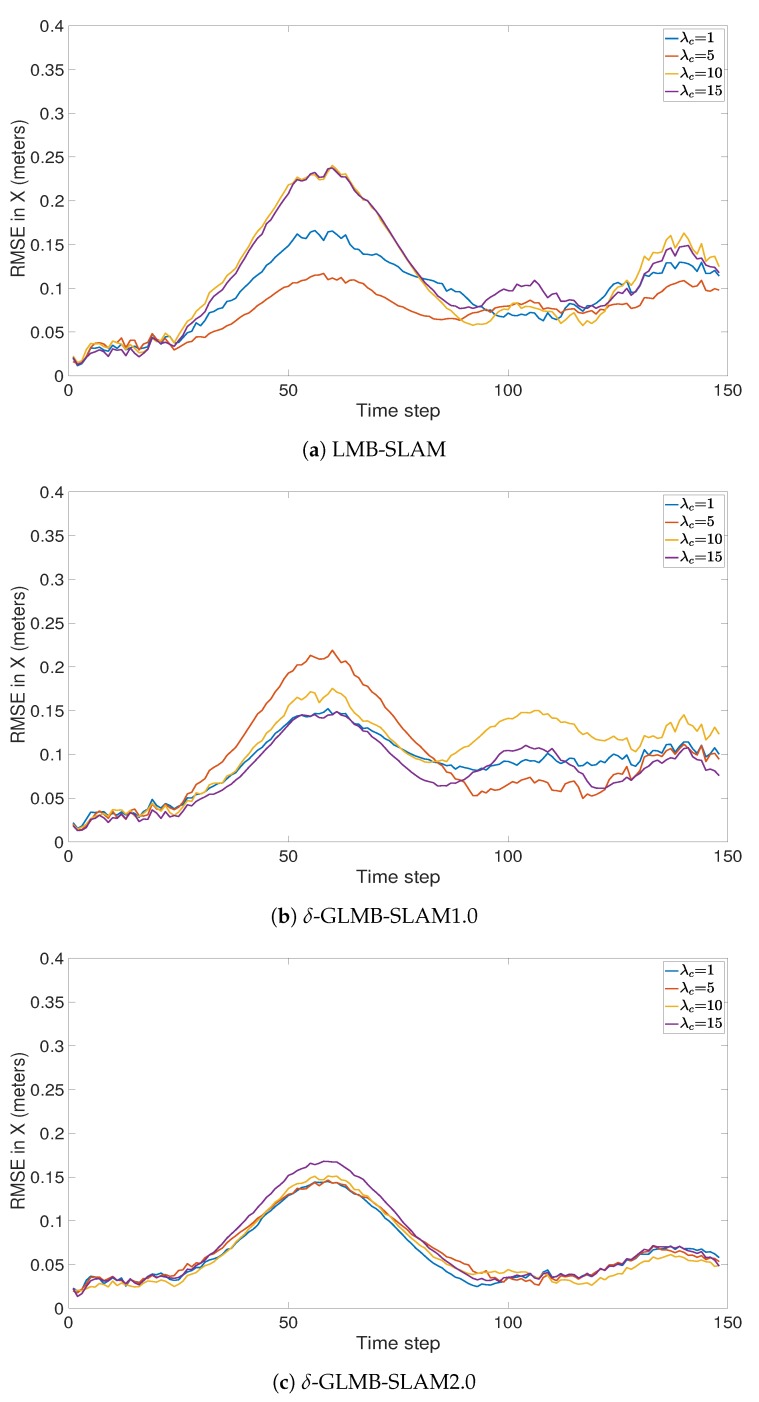
Comparison of RMSE in the X direction under varying clutter rates.

**Figure 3:**
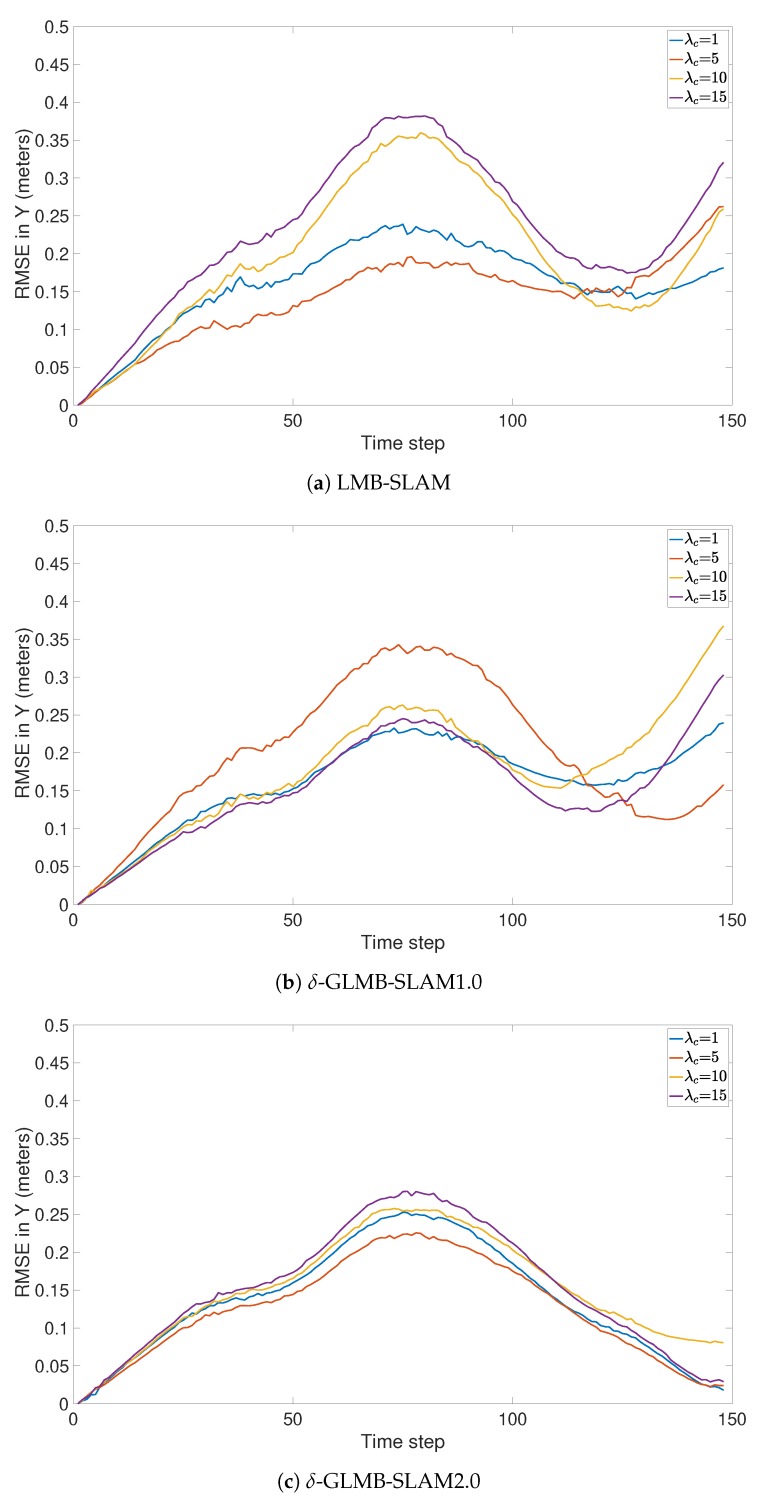
Comparison of RMSE in the Y direction under varying clutter rates.

**Figure 4:**
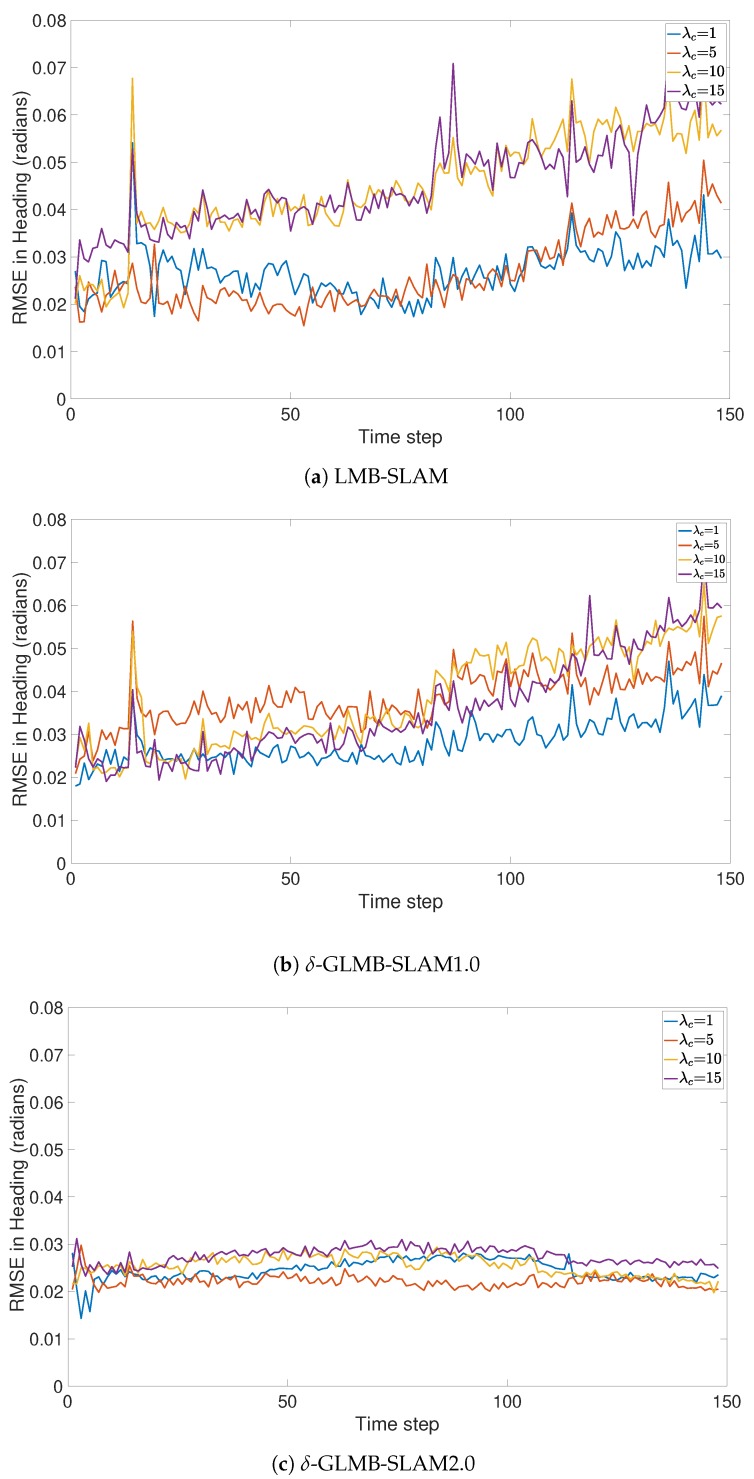
Comparison of RMSE in the heading under varying clutter rates.

**Figure 5:**
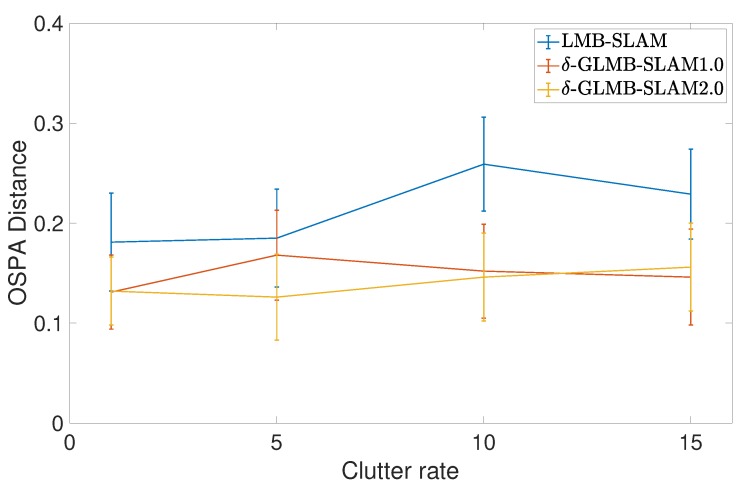
Comparison of the average Optimal Sub-Pattern Assignment (OSPA) distance with standard deviation under varying clutter rates. OSPA cut-off c = 0.5 and order p = 1.

**Figure 6:**
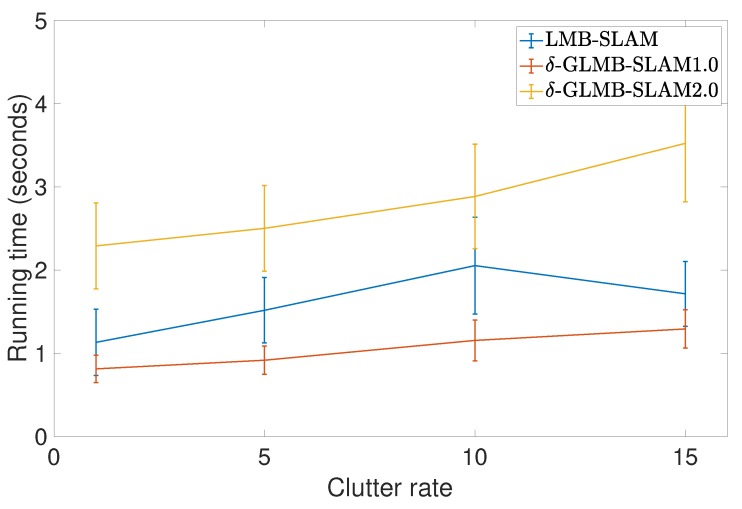
Comparison of average running time with standard deviation per time step under varying clutter rates.

All three algorithms were executed with 15 Monte Carlo (MC) runs per each clutter rate using 10 particles. Note that, in general, a larger number of particles produced a larger number of possible pose hypotheses during sampling, which yielded improved results in all three algorithms, albeit at a higher computational cost. However, a smaller number of particles was chosen to better evaluate and compare the improvements that can be gained by the proposed optimal kernel based sampling process. The estimated and actual robot trajectory of a sample MC run under each clutter rate is shown in Figure 1. It can be seen that δ-GLMB-SLAM2.0 produced superior results compared to LMB-SLAM and δ-GLMB-SLAM1.0 in both low and high clutter conditions and the performance of LMB-SLAM was inferior compared to δ-GLMB-SLAM1.0 and δ-GLMB-SLAM2.0. It can also be seen that LMB-SLAM produced more false features (red circles) compared to both δ-GLMB-SLAM1.0 and δ-GLMB-SLAM2.0 at high clutter rates. The root mean squared (RMS) robot pose estimation error in X, Y, and heading angle are compared in Figure 2, Figure 3 and Figure 4, respectively. It can be seen that δ-GLMB-SLAM2.0 produced smaller RMS errors in X and heading compared to δ-GLMB-SLAM1.0 and LMB-SLAM at all clutter rates. Furthermore, it can be seen that both LMB-SLAM and δ-GLMB-SLAM1.0 produced inferior results during re-visits to previously mapped areas (loop closure) from time steps 125 to 150 as the clutter rate increased, yielding significant drifts in the Y coordinate and heading angle. δ-GLMB-SLAM2.0 produced the smallest errors due to the improved sampling of pose particles using features from previously mapped areas. A comparison of the estimated feature map with the actual ground truth map is made in terms of the average Optimal Sub-Pattern Assignment (OSPA) distance in Figure 5. Clearly, both δ-GLMB-SLAM1.0 and δ-GLMB-SLAM2.0 outperformed LMB-SLAM. The average run times (i.e. the average times taken by each algorithm to calculate the trajectory and perform a map update at each time step) are compared in Figure 6, and it can be seen that δ-GLMB-SLAM2.0 required the highest run time, while δ-GLMB-SLAM1.0 the lowest run time under all clutter rates. This was consistent with the fact that the improved particle filter in δ-GLMB-SLAM2.0 required additional computations to sample a robot pose compared to δ-GLMB-SLAM1.0 and LMB-SLAM. It was also clear that the run time increased with the clutter rate in both LMB-SLAM and δ-GLMB-SLAM2.0 compared to δ-GLMB-SLAM1.0. The larger run time of LMB-SLAM with respect to δ-GLMB-SLAM1.0 was consistent with the fact that LMB-SLAM expanded its LMB distribution into a δ-GLMB distribution prior to measurement update, and combined the resultant hypotheses after the update step. In comparison, both δ-GLMB-SLAM1.0 and δ-GLMB-SLAM2.0 retained the hypotheses until further measurement updates invalidated insignificant hypotheses, which resulted in a significantly more robust performance in terms of pose estimation accuracy and OSPA distance compared to LMB-SLAM.

### 4.2. Real Results

**Figure 7:**
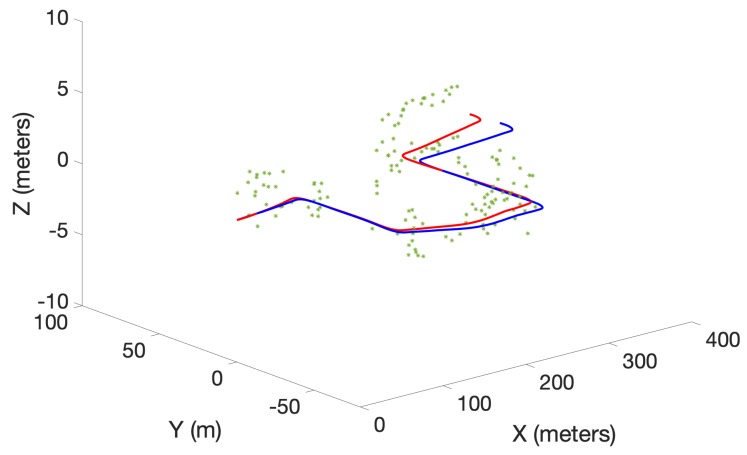
The estimated map (green) and the trajectory (red) using LMB-SLAM with ground truth trajectory (blue).

**Figure 8:**
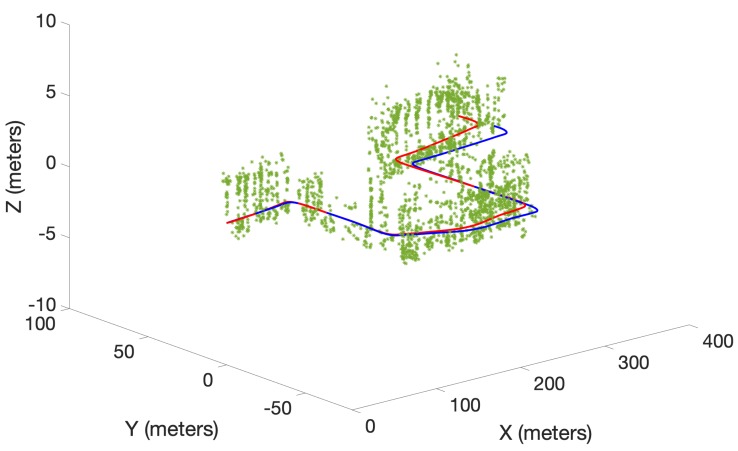
The estimated map (green) and the trajectory (red) using δ-GLMB-SLAM1.0 with ground truth trajectory (blue).

**Figure 9:**
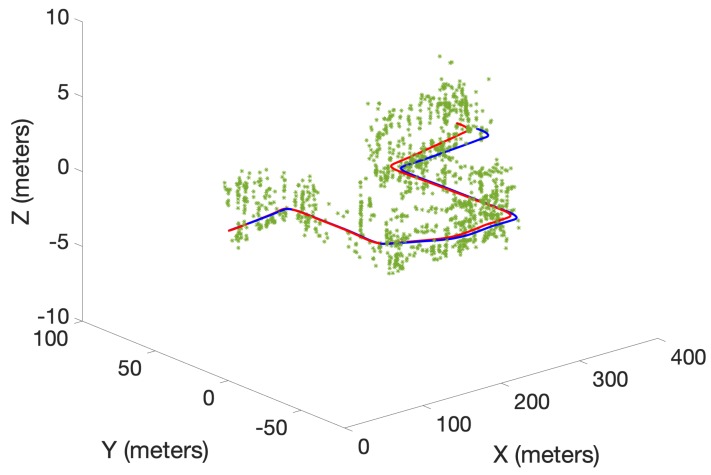
The estimated map (green) and the trajectory (red) using δ-GLMB-SLAM2.0 with ground truth trajectory (blue).

A preliminary experiment to test the performance of the proposed algorithm was also conducted using a real data set obtained from the Karlsruhe Institute of Technology and Toyota Technological Institute (KITTI) repository [29]. The KITTI repository contains several sequences of datasets collected from a moving car fitted with several sensors including stereo cameras. In this work, a portion of the odometry sequence 00 was used. Oriented FAST and rotated BRIEF (ORB) features [30] extracted from the stereo image sequence of the cameras were triangulated into 3D and used as measurements. The maximum observable range was limited to 20 m. Further, odometry was obtained by executing the viso2 library package, which provides visual odometry [31]. Linear and angular velocities in 2D were used as the control inputs for the three algorithms. Unlike in the simulated experiment, the number of detected ORB features dramatically increased as the robot moved in the real environment. Therefore, to achieve a tractable running time over several MC runs, a smaller number of trajectory particles (5) was chosen. This lower number of particles was found to be sufficient to demonstrate the superior performance due to the proposed optimal kernel-based sampling process in δ-GLMB-SLAM2.0, with respect to visual odometry, LMB-SLAM and δ-GLMB-SLAM1.0. The estimated map and the robot trajectories are shown in Figure 7, Figure 8 and Figure 9.

**Figure 10:**
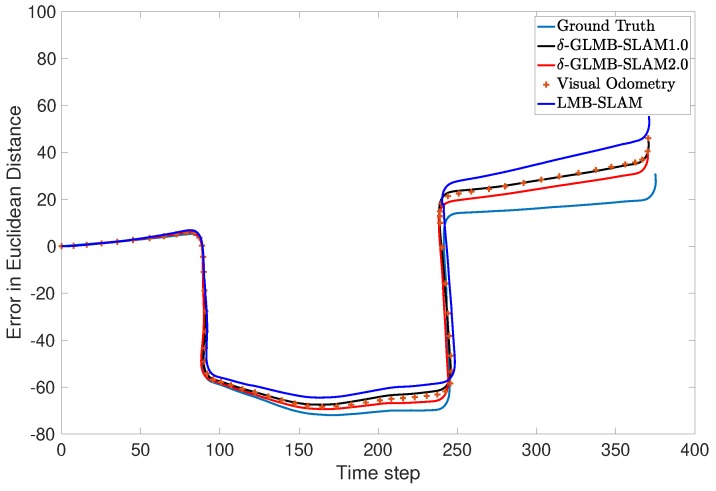
Comparison of the estimated robot trajectories and visual odometry with respect to the ground truth.

**Figure 11:**
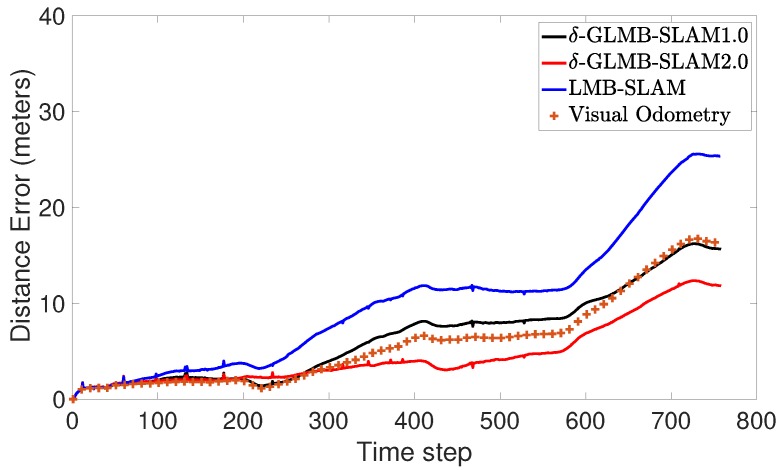
Comparison of the Euclidean distance error between the estimated and ground truth trajectories verses time step.

Note that, unlike in the simulated results, it is not possible to show the mapping error in the real experiment. This is because the feature type used (ORB) is a non-semantic, mathematical feature and no ground truth information regarding the true number and location of these features is provided with the KITTI dataset. Therefore, as is the case with most publicly available dataset-based SLAM experiments, the only performance measure possible was to compare estimated and ground truth trajectories. A comparison of the estimated trajectories and visual odometry with ground truth is shown in Figure 10. Furthermore, a comparison of the Euclidean distance errors among ground truth, the estimated trajectories and visual odometry are shown in Figure 11. It can be seen that δ-GLMB-SLAM2.0 produced the smallest deviation from ground truth when compared with LMB-SLAM and δ-GLMB-SLAM1.0. δ-GLMB-SLAM2.0 even outperformed visual odometry despite the small number of particles used. The estimated trajectory of LMB-SLAM had the largest deviation from the ground truth, producing inferior results, even when compared to visual odometry. The map estimate produced by LMB-SLAM (Figure 7) yielded a significantly lower number of features compared to δ-GLMB-SLAM1.0 and δ-GLMB-SLAM2.0 and requires a considerable amount of fine tuning of the parameters.

## 5. Conclusions

In this paper, we have presented a new RFS-based SLAM algorithm referred to as δ-GLMB-SLAM2.0. Similar to earlier RFS-based SLAM algorithms, δ-GLMB-SLAM2.0 factorizes the SLAM posterior into the landmark map posterior and robot trajectory posterior via Rao–Blackwellization. However, instead of using a standard particle filter, the robot trajectory is propagated using an optimal kernel based particle filter, and the landmark map is estimated using an efficient variant of the δ-GLMB filter based on a Gibbs sampler. The performance of δ-GLMB-SLAM2.0 was evaluated using a series of simulations and a real experiment, and compared to its predecessor δ-GLMB-SLAM1.0 and the LMB-SLAM algorithm with a Gibbs sampling-based joint map prediction and update approach. From the simulated and the real results, it can be seen that the proposed δ-GLMB-SLAM2.0 algorithm outperforms both δ-GLMB-SLAM1.0 and LMB-SLAM in terms of pose estimation error and the quality of the map under varying clutter conditions while requiring slightly higher computational times. The higher quality in the pose and the map estimation error can be attributed to two factors. First, the sampling of the particle filter no longer relies only on the control signals but instead uses the controls, measurements and prior map in order to produce a set of highly likely robot poses during the robot pose sampling step. Second, both δ-GLMB filters maintain multiple hypotheses for the landmark map state and remove insignificant hypotheses as further measurements invalidate contradicting hypotheses. Although δ-GLMB-SLAM1.0 also relies on the δ-GLMB filter for landmark map estimation, an improved particle filter results in better estimation performance in δ-GLMB-SLAM2.0. On the other hand, the LMB-SLAM algorithm combines multiple hypotheses during the measurement update step into a single LMB distribution with loss of information. As a result, drifts in landmark map estimates are visible during re-visits to previously mapped areas, which in turn result in drifts of the robot pose estimate. The higher computational time of δ-GLMB-SLAM2.0 to that of δ-GLMB-SLAM1.0 is due to additional computations required during the particle filter sampling step. Furthermore, it can be seen that standard measurement gating, particle level parallelization, and the Gibbs sampler-based hypothesis generation, result in a lower computational time of δ-GLMB-SLAM1.0 compared to LMB-SLAM, which outweighs the savings expected by combining multiple hypotheses.

## Figures and Tables

**Table 1:** Parameters used in the simulation.

Robot/Sensor Parameters	Variable	Values
Velocity	*v*	1 m/s
Sensor FOV	Range (*r*)	0 to 3 m
	Bearing (*b*)	−π to +π
Control Noise	Velocity ($\sigma_{v}$)	0.1 m/s
	Steering Angle ($\sigma_{a}$)	$2^{0}$
Measurement Noise	Range ($\sigma_{r}$)	0.05 m
	Bearing ($\sigma_{b}$)	$0.5^{0}$

## Abbreviations

The following abbreviations are used in this manuscript:

EKF	Extended Kalman Filter
MAP	Maximum a Posteriori
SLAM	Simultaneous Localization and Mapping
RFS	Random Finite Set
FISST	Finite Set Statistics
PHD	Probability Hypothesis Density
CPHD	Cardinalized Probability Hypothesis Density
LMB	Labeled Multi-Bernoulli
GLMB	Generalized Labeled Multi-Bernoulli

## References

[1] Smith R.C., Cheeseman P. (1986). On the Representation and Estimation of Spatial Uncertainty. Int. J. Robot. Res..

[2] Dissanayake M.W.M.G., Newman P., Clark S., Durrant-Whyte H.F., Csorba M. (2001). A solution to the simultaneous localization and map building (SLAM) problem. IEEE Trans. Robot. Autom..

[3] Montemerlo M., Thrun S., Koller D., Wegbreit B. FastSLAM: A factored solution to the simultaneous localization and mapping problem. Proceedings of the AAAI National Conference on Artificial Intelligence.

[4] Thrun S., Liu Y., Koller D., Ng A.Y., Ghahramani Z., Durrant-Whyte H. (2004). Simultaneous localization and mapping with sparse extended information filters. Int. J. Robot. Res..

[5] Kschischang F.R., Frey B.J., Loeliger H.A. (2001). Factor graphs and the sum-product algorithm. IEEE Trans. Inf. Theory.

[6] Thrun S., Montemerlo M. (2006). The graph SLAM algorithm with applications to large-scale mapping of urban structures. Int. J. Robot. Res..

[7] Dellaert F., Kaess M. (2006). Square Root SAM—Simultaneous Localization and Mapping via Square Root Information Smoothing. Int. J. Robot. Res..

[8] Cadena C., Carlone L., Carrillo H., Latif Y., Scaramuzza D., Neira J., Reid I., Leonard J.J. (2016). Past, Present, and Future of Simultaneous Localization and Mapping: Toward the Robust-Perception Age. IEEE Trans. Robot..

[9] Mahler R. (2007). Statistical Multisource-Multitarget Information Fusion.

[10] Mahler R.P.S. (2003). Multitarget bayes filtering via first-order multitarget moments. IEEE Trans. Aerosp. Electron. Syst..

[11] Mahler R. (2007). PHD filters of higher order in target number. IEEE Trans. Aerosp. Electron. Syst..

[12] Vo B.T., Vo B.N. (2013). Labeled random finite sets and multi-object conjugate priors. IEEE Trans. Signal Process..

[13] Vo B.N., Vo B.T., Phung D. (2014). Labeled Random Finite Sets and the Bayes Multi-Target Tracking Filter. IEEE Trans. Signal Process..

[14] Reuter S., Vo B.T., Vo B.N., Dietmayer K. (2014). The Labeled Multi-Bernoulli Filter. IEEE Trans. Signal Process..

[15] Vo B.N., Vo B.T., Hoang H.G. (2017). An Efficient Implementation of the Generalized Labeled Multi-Bernoulli Filter. IEEE Trans. Signal Process..

[16] Mullane J., Vo B.N., Adams M.D., Wijesoma W.S. A random set formulation for Bayesian SLAM. Proceedings of the 2008 IEEE/RSJ International Conference on Intelligent Robots and Systems.

[17] Mullane J., Vo B.N., Adams M.D. Rao-Blackwellised PHD SLAM. Proceedings of the 2016 IEEE International Conference on Robotics and Automation (ICRA).

[18] Mullane J., Vo B.N., Adams M.D., Vo B.T. (2011). A Random-Finite-Set Approach to Bayesian SLAM. IEEE Trans. Robot..

[19] Lee C.S., Clark D.E., Salvi J. (2012). SLAM with single cluster PHD filters. Proceedings of the 2016 IEEE International Conference on Robotics and Automation (ICRA).

[20] Deusch H., Reuter S., Dietmayer K. (2015). The Labeled Multi-Bernoulli SLAM Filter. IEEE Signal Process. Lett..

[21] Moratuwage D., Vo B.N., Wang D. Collaborative Multi-vehicle SLAM with moving object tracking. Proceedings of the 2013 IEEE International Conference on Robotics and Automation (ICRA).

[22] Reuter S. (2014). Multi-Object Tracking Using Random Finite Sets. Ph.D. Thesis.

[23] Montemerlo M., Thrun S., Roller D., Wegbreit B. FastSLAM 2.0: An improved particle filtering algorithm for simultaneous localization and mapping that provably converges. Proceedings of the IJCAI International Joint Conference on Artificial Intelligence.

[24] Montemerlo M., Thrun S. (2007). FastSLAM: A Scalable Method for the Simultaneous Localization and Mapping Problem in Robotics.

[25] Cappe O., Godsill S.J., Moulines E. (2007). An overview of existing methods and recent advances in sequential Monte Carlo. Proc. IEEE.

[26] Moratuwage D., Adams M., Inostroza F. *δ*-Generalised Labelled Multi-Bernoulli Simultaneous Localisation and Mapping. Proceedings of the 2018 International Conference on Control, Automation and Information Sciences (ICCAIS).

[27] Doucet A., Johansen A.M. (2011). A tutorial on particle filtering and smoothing: Fifteen years later. The Oxford Handbook of Nonlinear Filtering.

[28] Reuter S., Danzer A., Stuebler M., Scheel A., Granström K. A fast implementation of the Labeled Multi-Bernoulli filter using gibbs sampling. Proceedings of the 2017 IEEE Intelligent Vehicles Symposium (IV).

[29] Geiger A., Lenz P., Stiller C., Urtasun R. (2013). Vision meets robotics: The KITTI dataset. Int. J. Robot. Res..

[30] Rublee E. ORB: An efficient alternative to SIFT or SURF. Proceedings of the IEEE International Conference on Computer Vision.

[31] Geiger A., Ziegler J., Stiller C. StereoScan: Dense 3D reconstruction in real-time. Proceedings of the 2011 IEEE Intelligent Vehicles Symposium (IV).

